# Optimization of Components with Topology Optimization for Direct Additive Manufacturing by DLMS

**DOI:** 10.3390/ma16155422

**Published:** 2023-08-02

**Authors:** Frantisek Sedlacek, Tomas Kalina, Martin Stepanek

**Affiliations:** Faculty of Mechanical Engineering, University of West Bohemia, 301 00 Plzen, Czech Republic; tkalina@fst.zcu.cz (T.K.); stepanem@fst.zcu.cz (M.S.)

**Keywords:** numerical simulation, topology optimization, additive manufacturing, L-PBF, design methodology, process simulation

## Abstract

This paper presents a novel design methodology that validates and utilizes the results of topology optimization as the final product shape. The proposed methodology aims to streamline the design process by eliminating the need for remodeling and minimizing printing errors through process simulation. It also eliminates the repeated export and import of data between software tools. The study includes a case study involving the steering column housing of a racing car, where Siemens NX Topology Optimization was used for optimization, and verification analysis was conducted using the NX Nastran solver. The final solution was fabricated using AlSi10Mg via direct metal laser sintering on a 3D printer and successfully validated under real conditions. In conclusion, this paper introduces a comprehensive design methodology for the direct utilization of topology optimization, which was validated through a case study, yielding positive results.

## 1. Introduction

Topology optimization is advantageous for manufacturers, as it determines the optimal material distribution in a design space based on required loads. Nowadays, topology optimization extends its applications to simulations in other technical fields, such as an article of Mr. Cao, who used a topology optimization for a next-generation wireless data center network [1]. However, a significant drawback is the need to remodel the structure for the final design. Structural optimization is a relatively old research field. Analytical and numerical techniques have long been exploited in the solution of structural design problems. In the 17th century, in his book *Discorsi e dimostrazioni matematiche*, Galileo Galilei introduced one of the first concepts about the optimal shape of structural elements [2]. His book was followed by several works by Gottfried W. Leibniz, who formulated the basis of analytic procedure. Maxwell’s works in 1870 introduced a theory to find the best material distribution through a principal stress field for civil engineering problems (bridges, etc.). In 1904, Anthony G. M. Michell continued Maxwell’s work to create optimal beam structures [3]. The use of structural optimization based on numerical techniques has seen significant growth with the coming of modern computing devices and materials [4]. Many types of structural optimization exist today, and they are directly implemented in commercial software [5]. Geometry (sizing) optimization [6], topometric optimization, topography optimization [7], shape optimization [8,9], freeform optimization, and topology optimization [10] are among the most well known.

The origins of topological optimization can be traced back to the early 1970s, when Prager and Rozvarny formulated their initial theory [11]. This optimization technique aims to determine the optimal distribution of structure within a predefined area. It employs a fixed number of finite elements and assigns a single input variable, known as the pseudo-density modulus, to each element of the resolved volume fraction. Topological optimization methods can generally be classified into three main categories: element-based, discrete, and combined approaches [10,12,13,14].

Solid isotropic microstructures with penalization (SIMP) [15,16], rational approximation of material properties (RAMP) [17], optimal microstructure with penalization (OMP) [18], non-optimal microstructures (NOM), and dual discrete programming (DDP) [19] are among the most used element-based methods of topology optimization. Evolutionary structural optimization (ESO) [20], bidirectional evolutionary structural optimization (BESO) [21], and additive evolutionary structural optimization (AESO) [22] are some of the most famous discrete methods of topology optimization. Extended finite-element method (XFEM) together with deformable simplicial complex (DSC) is a combined approach for solving topology optimization.

Additive manufacturing technology, also referred to as 3D printing, has been in existence for several decades. However, in recent years, it has sparked a manufacturing revolution. This process involves the joining of materials to create objects based on 3D model data, typically layer by layer [23]. Initially, additive manufacturing was primarily used for producing visual prototypes, test samples, and educational purposes [24]. However, over time, it has evolved into an advanced technique capable of manufacturing end-user products [25] across various industries [26,27], including aviation, motorsports, trains, medicine, and more [28]. The widespread adoption of additive manufacturing has revolutionized manufacturing processes, enabling the production of complex and customized products with significant implications for various sectors.

In response to the significant growth and widespread adoption of additive manufacturing technology in recent years, the International Organization for Standardization (ISO) and the American Society for Testing and Materials (ASTM) have developed multiple standards. These standards encompass general principles, terminology [29,30], and specific guidelines for design [31,32]. These efforts aim to establish a unified framework and ensure consistency in the application of additive manufacturing across various industries.

The ISO/ASTM 52900-15 standard [33] is considered a foundational document in the field of additive manufacturing. It offers a fundamental framework for classifying additive manufacturing technologies, specifically by dividing them into seven main categories based on the additive manufacturing process: (VAT, SLA, DLP, and CDLP) photopolymerization [34,35], material extrusion [36,37], material jetting [38,39], binder jetting [40,41], powder bed fusion [42,43,44,45], directed energy deposition [46,47], and sheet lamination [48].

In this case, the powder bed fusion–additive manufacturing (AM) process, specifically direct metal laser sintering (DMLS), was utilized. This process was pioneered by EOS (Electro Optical System, Krailling, Germany) in 1994, becoming the first commercially viable method for producing metal parts through AM. The underlying principle of this technology involves the selective melting of thin layers (20–60 µm) of metal powder using a laser or electron beam. The build platform is used to construct parts layer by layer, with a relatively high level of geometric precision (±0.05 mm). The metal powder is deposited onto the build platform either through a re-coater arm or a roller from the powder supply. L-PBF additive technology is suitable for producing prototypes and one-off production, as demonstrated by Rokicki and Rahmati in their papers [49,50]. Indeed, L-PBF can be utilized for fabricating structures with lattice structures. Gupta, in his work, mentioned the damping effects of this structure, which can be advantageous, for example, for dampening vibrations transmitted into the steering wheel of a student formula car. This can improve the overall performance and handling of the vehicle [51].

It is common practice that the resulting geometry from topology optimization needs to be remodeled before verification and printing. Prathyusha and Budu applied a methodology that involved topology optimization for a landing door bracket. However, during the process, it was necessary to perform remodeling after the initial topology optimization [52,53]. Similarly, Dienemann employed topological optimization for battery-pack cooling for forced convection based on Darcy flow. However, the efficiency of its design was also compromised due to the need for remodeling the outcome of topological optimization [54]. Barbieri contrasted design methodologies by utilizing generative design and topology optimization tools. Although the generative design yielded printable outcomes, his approach overlooked manufacturability evaluation and the potential errors that may arise during the manufacturing process [55]. The objective of this research is to establish a fundamental methodology for the direct utilization of topology optimization outcomes in additive manufacturing, specifically laser powder bed fusion (L-PBF). The proposed methodology aims to address the challenges associated with preserving the integrity of the optimized structure during the additive manufacturing process while also compensating for errors and limitations inherent in the AM process, such as self-supporting structures and distortion compensation. This research seeks to integrate various aspects that are typically addressed separately in the existing literature, including the influence of support placement and deformation induced by thermal loading [56]. Kim investigated the influence of the placement, type, and size of supports on part deformation in his article. This research served as a basis for simulating support placement and was extended to incorporate compensatory deformations into the CAD model, aiming to achieve a more accurately manufactured component [56]. In his article, Bouabbou assessed the influence of residual powder around the printed part on heat transfer and the occurrence of printing errors. The simulation of the manufacturing process presented in this study takes into consideration the thermal properties of the residual powder for heat transfer analysis [57]. By combining these activities into a unified workflow, the proposed methodology aims to enhance the overall efficiency and effectiveness of the topology optimization and additive manufacturing process. The housing of the steering column of a racing car was used as the case study for determining the basic methodology of the “rapid prototyping” process.

### Current Solution of Case Study

**Figure 1:**
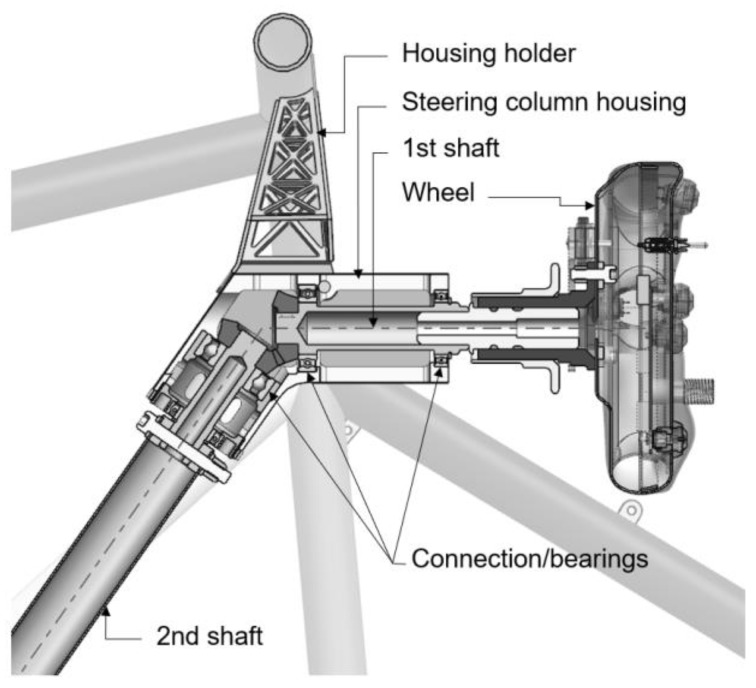
Schematic of current solution of steering column housing and connected components.

The current solution of the steering column housing is a CNC-machined part made from aluminum alloy (EN AW 7075). This part is connected to the frame of the car using a steel (SR355) weldment holder. The layout of the housing with all connected components (internal axes, gears, bearings, etc.) is shown in Figure 1. The weight of the current solution of the steering column housing and the holder is 0.514 kg.

## 2. Optimization of the Steering Column Housing

### 2.1. Material

In spite of progress in the case of developing metal composite materials [58], EOS aluminum AlSi10Mg [59,60] (Light Metal for Motorsports and Aerospace Interior Applications, provided by the EOS GmbH Electro Optical Systems in the form of a gas-atomized metal powder) was set as the material of the structure. This alloy provides a favorable price-to-performance ratio, rendering it a cost-effective selection, and it is widely used for motorsports and aerospace applications [56,61]. The chemical composition of the material is given in Table 1.

The experimental measurements of the material AlSi10Mg were taken for determination of the mechanical properties. Specimen geometry and the testing method for tensile test were chosen according to the ASTM E8/E8M-16a standard [62]. The specimens were fabricated on the print bed in two main positions: horizontally and vertically to the print bed, using the EOS M 290 machine [63,64]. The process parameters used for fabrication of the AlSi10Mg specimens are given in Table 2.

**Figure 2:**
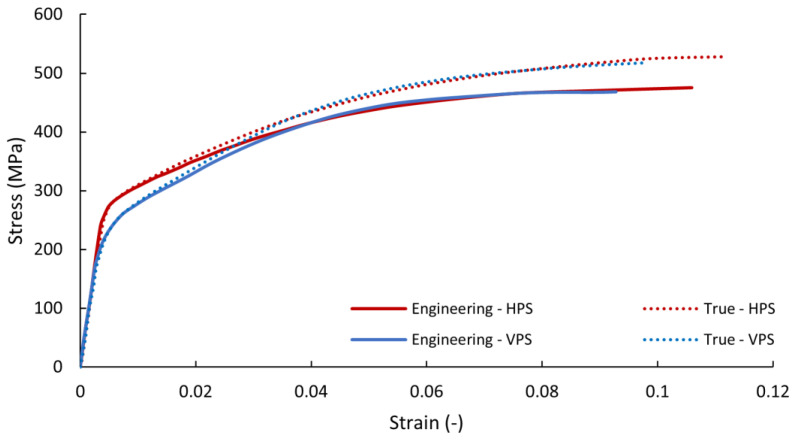
Engineering and true stress–strain curves of vertically and horizontally printed AlSi10Mg specimens.

A Zwick-Roell Z050 electro-mechanical testing machine with a 5 kN load cell and 20 Hz sampling frequency was used for uniaxial tensile tests. The stress–strain curves of the vertically and horizontally printed specimens are given in Figure 2.

The mechanical properties of the material are summarized in Table 3.

### 2.2. Design Area of Optimization

**Figure 3:**
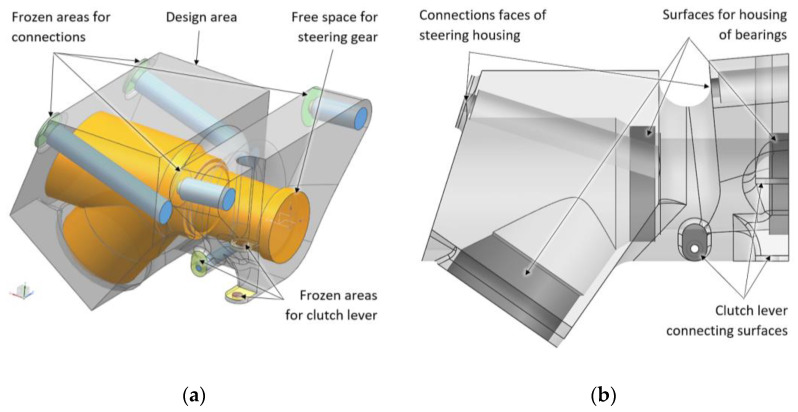
(**a**) Three-dimensional model of boundary volume for topology optimization; (**b**) frozen areas of topology optimization.

The Siemens NX1888 Topology Optimization for Designers software (Siemens NX 12.0.2) was used for optimization. The volume that represents the mounting boundaries where the final structure may be was created. The space for connecting the related components, bolts for attaching the steering column housing to the frame, and the necessary space for the tools were subtracted from this volume. The final model of the design area for topology optimization is given in Figure 3.

### 2.3. Parameters of Topology Optimization

The minimization of the strain energy subject to the mass target (minimalizing compliance) of the structure was chosen as the objective function of the optimization. The mass target of the final structure, *C_m_* = 0.29 kg, was set as the design constraint. A 2.5 mm input mesh resolution was used for the optimization. Material spreading of 70% and self-supporting with an angle to the base plate of 45% were used as manufacturing constraints. Frozen areas (areas where optimization cannot remove material) were created with the definition of the minimum thickness of the walls (2.5 mm for surfaces that are intended for connecting the steering column housing and 3 mm for cylindrical faces that are intended for housing the bearings of the main shafts of the steering gear) (see Figure 3).

### 2.4. Specification of Input Load Cases

Obtaining the loading condition directly from real measurements is the optimal approach [65]. Together, the four most critical load cases were used for structural analysis as the input for topology optimization (according to critical driving conditions). The first load case was cornering (radial forces in transverse direction: *F_RtI_* = 2153 N for the cylindrical face of the housing of the first shaft bearing; *F_RtII_* = −4819 N for the second housing of the bearing; and moment *M_tIII_* = 132 Nm). The second load case was deceleration (axial force *F_Ad_* = −1890 N). The third one was acceleration (axial force *F_Aa_* = −1610 N) and their combination (cornering with deceleration). The individual load cases were calculated analytically using data from the data logger of the telemetry from a real race (G-G diagram).

**Figure 4:**
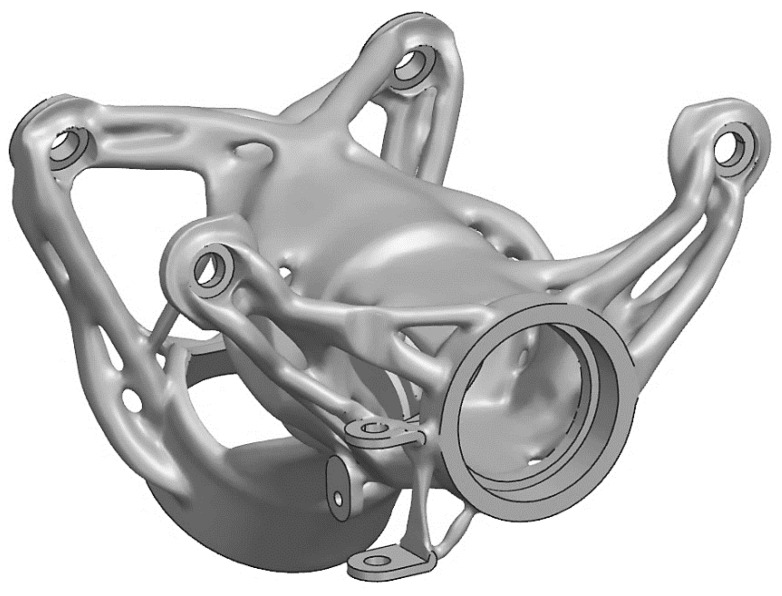
The final structure of the topology optimization.

The final solution with a mass of 0.288 kg was found after 49 optimization cycles. Automatic smoothing of the results of the optimized structure (so-called normalized material) is a great advantage of this optimization solver (by the associated postprocessor). The results of the final structure are given in Figure 4.

## 3. Verification Analysis of the Result of TO

### 3.1. Generation of FE Mesh from STL Model

The final structure obtained from the topology optimization is in the stereolithography file format (STL). An STL format describes the surface of a structure or a solid object using the unit normal and vertices of unstructured triangles. This format approximates the 3D surfaces of a solid model with oriented triangles (facets) of different sizes and shapes (aspect ratio) in order to achieve a sufficiently fine resolution to achieve the required quality and tolerance of the surface of the structure [66]. This format is otherwise directly applicable to 3D printing, but it is difficult to perform on STL discretization using the finite-element method for verification analysis. Using an STL directly to create a volumetric 3D FEM mesh is supported by only a few FEM pre-processors and has many limitations—such as the possibility of using only the first-order elements, etc. A polygon model created by remodeling an STL structure by using conventional CAD methods is the most common way, but it is very time-consuming.

One of the new methods is the so-called convergent model that eliminates the necessity for remodeling. Convergent-type solid or sheet bodies are the faceted representation of geometry in which edges and faces are based on tessellation rather than on parameterization. Convergent-type CAD geometry is typically generated automatically by special modeling features of reverse-engineering modules. A closed surface of the STL body without mesh errors is the only condition for creating a convergent model. 

**Figure 5:**
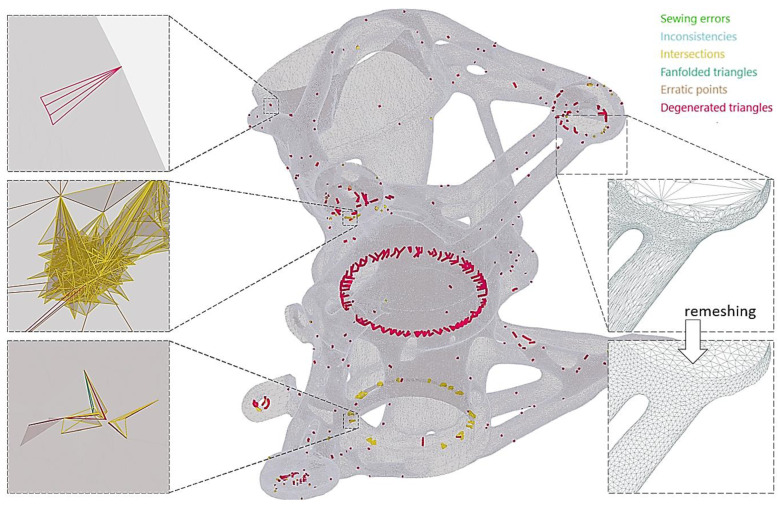
Main types of STL errors.

Intersections, degenerated triangles, erratic points, inconsistencies, fanfolded triangles, isolated points, and edges are the most common mesh errors. Intersections mean that one or more triangles penetrate another one. Degenerated triangles are triangles of the STL surface that are too small (their ratio of height × length is less than 1 × 20). Erratic points are, for example, if two holes touch each other. Inconsistencies exist if triangles of the STL surface having opposite surface normals border on each other. Fanfolded triangles of the surface partly lie on top of each other and are connected to each other on at least one side. Isolated points and edges are features that do not belong to any triangle of the mesh and are located separately. All mesh errors of the STL were corrected using GOM Inspect 2016 software. Overall, 5092 mesh errors were detected and repaired using the Eliminate Mesh Errors function (see Figure 5). 

### 3.2. Verification Using Independent Structural Analysis

**Figure 6:**
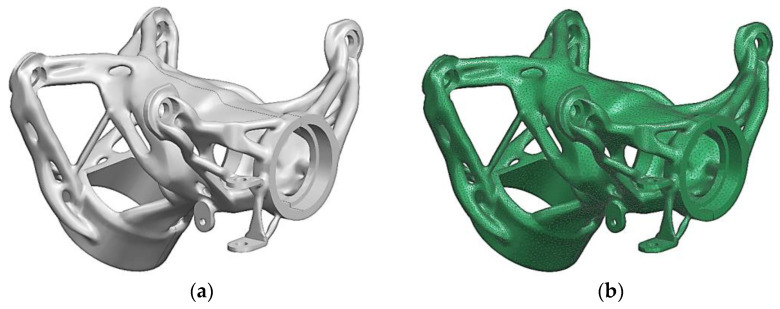
(**a**) Generated convergence model; (**b**) FEM mesh for verification analysis.

The final structure obtained from the topology optimization and repaired in GOM Inspect 2016 software was imported into the CAD module of the Siemens NX 1888 software, and it was translated to a convergence (polygon) model to allow the application of the FEM mesh. Siemens NX Simcenter 3D 1888 software was used as the pre-processor of the FEM analysis [67]. The 3D FEM mesh with tetrahedral second-order elements with the relative size of the edge 3 mm was applied on the convergence body (see Figure 6).

**Figure 7:**
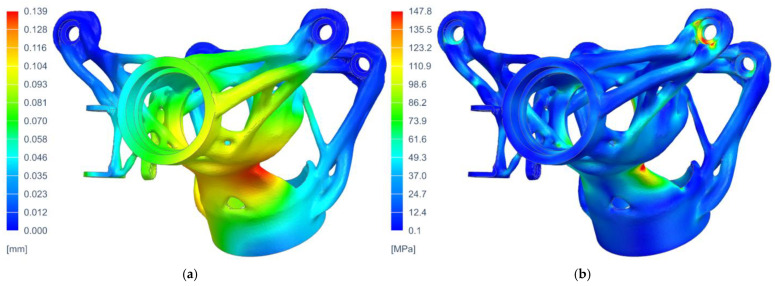
The results of structural analysis of the steering column housing for the most critical load case: (**a**) displacement—magnitude (m); (**b**) equivalent tensile stress—according to Von Mises (MPa).

The boundary conditions were taken from the optimization analysis, and the NX Nastran—SOL 106 Static Nonlinear Solver was used. The mechanical properties of the material were defined according to the parameters obtained by experimental measurements given in Figure 2 and Table 3. The results of the displacement and equivalent stress (according to Von Mises) for the most critical load case (the combination of deceleration and cornering) are given in Figure 7. The displacement results showed sufficient stiffness of the overall structure, and the values of the reduced stress did not exceed the required safety of the structure, i.e., k = 1.25.

## 4. Functional Specimen

### 4.1. Pre-Processing of Functional Specimen Manufacturability

The manufacturability of the final structure using L-PBF was analyzed prior to the fabrication process in the AM module of Siemens NX 1888 software. The analysis included checks for the printable volume to ensure the model was within the acceptable range for AM. Additionally, the minimum radii of concave and convex curvature (with a specific radius value of *r_c_* = 0.5 mm), overhangs [68], and minimum wall thickness were assessed. Maintaining an appropriate wall thickness (recommended value of *t_w_* = 0.4 mm) is crucial to prevent distortion caused by the high temperatures involved in the process.

**Figure 8:**
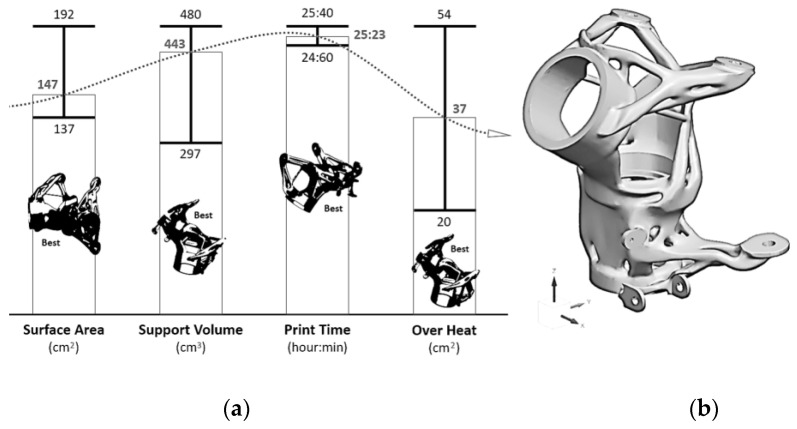
Optimization of orientation of part relative to printing bed, (**a**) results of individual optimization categories (minimum, maximum and selected), (**b**) resulting optimized part position.

The orientation of the part relative to the printing bed is a crucial parameter (lines 65–66). The part orientation was optimized using Siemens NX 1888 Additive Manufacturing software. The four main parameters in the optimization process (surface area, print time, support volume [69,70,71], and overheating) were considered. The weighting method was used in the optimization process. In this case, overheating and surface area were selected as priority parameters. The final part orientation was found to have a surface area of 187 cm^2^ (i.e., 40% of the total), overheating of 33 cm^2^ (i.e., 7% of the total), and support volume of 336 cm^3^. (The area needing support was 74.1 cm^2^, i.e., 16% of the total.) The most favorable orientation for individual parameters, including the ranges of individual values, and the final results are given at Figure 8.

**Figure 9:**
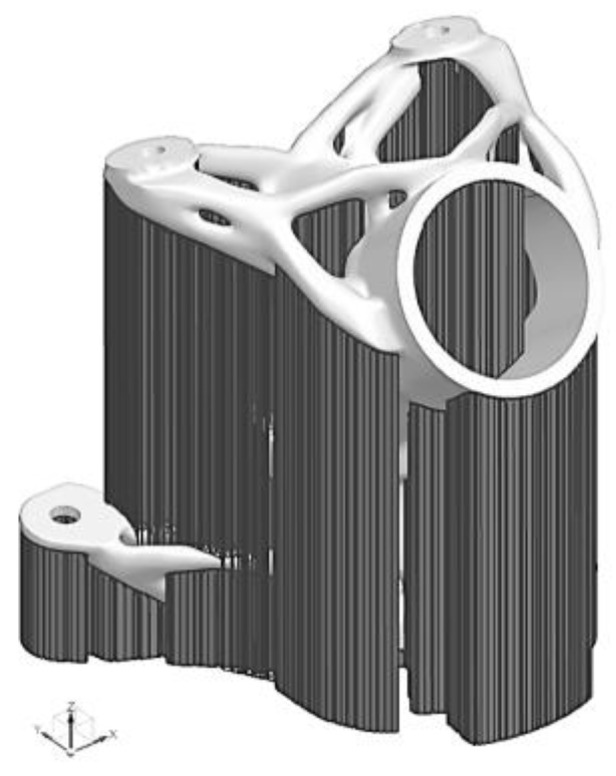
Final orientation of model with generated block supports for AM fabrication.

The orientation of the part with generated block supports (with critical angle 45° and gap between part and bed 8 mm) is given in Figure 9.

### 4.2. Numerical Simulation of Printing Process

The L-PBF printing process generates high temperatures, which can result in various issues such as local overheating, geometric distortion, shrink lines, and even printing interruptions caused by collisions between the re-coater and the fabricated part. To address these challenges, numerical simulation of the additive manufacturing process proves valuable, as it enables the prediction of potential issues and provides opportunities for compensation or complete elimination of these problems. By leveraging numerical simulation, it becomes possible to proactively mitigate the impact of residual heat and optimize the printing process for enhanced quality and performance.

In this case, the Simcenter™ 3D 1888 Additive Manufacturing software was employed. This specialized module utilizes a digital twin of the printed part, encompassing the build tray, residual powder, and support structures, to simulate the direct metal laser sintering process. The software employs a finite-element method-based solver, providing a significantly higher precision representation of the simulated part compared to conventional solvers that employ coarse voxel meshes.

In the first stage, the block supports were re-generated to simulation supports with homogenized orthotropic mechanical properties. The simulation in this process consists of two interconnected analyses (thermal computed with Simcenter Samcef Thermal—Heat transfer solver and mechanical computed with Simcenter Samcef—Nonlinear Analysis solver). The finite-element models (FEM) were created for both simulations, including the printing powder and slicing of printed parts. 

**Figure 10:**
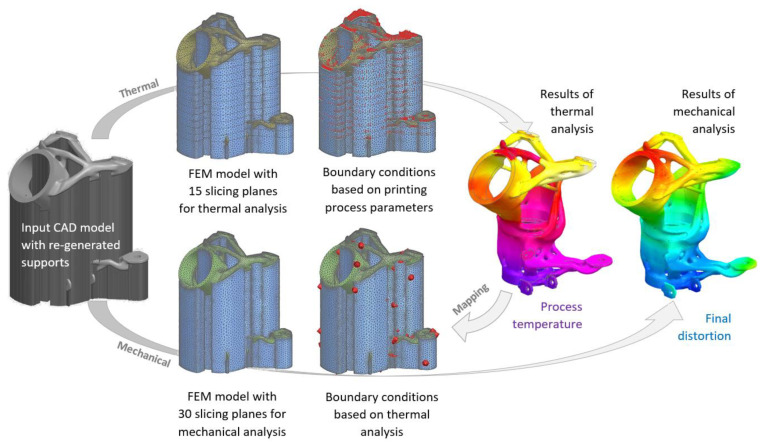
Flowchart of numerical simulation of printing process.

The thermal FEM was created from first-order tetrahedral CTETRA4 elements with a relative element size of 1.8 mm (950,582 elements) and 15 slicing planes for boundary conditions. The mechanical FEM was created from the same CTETRA4 elements with a relative element size of 1.5 mm (1,043,392 elements) and 30 slicing planes for boundary conditions. The thermal and mechanical FEM models used are shown in Figure 10.

The boundary conditions (heat loads, radiation, and convection) in the thermal simulation were automatically generated for individual slicing planes by the pre-processor based on the defined process parameters for printing (recoating time, base plate and ambient temperature, heat transfer coefficient of the ambient gas, alpha of the build plate, laser power, etc.). The mainly used process/thermal parameters are given in Table 4. The same process parameters as for the production of test specimens (see Table 2) were used for numerical simulation.

**Figure 11:**
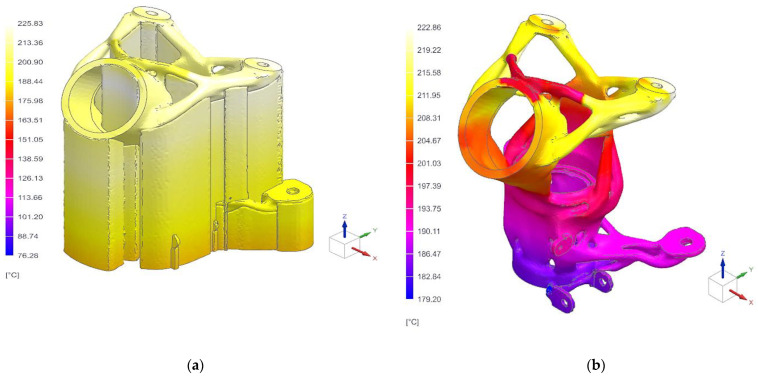
Thermal analysis results of printed specimen: (**a**) specimen with supports; (**b**) specimen after support removal.

The final printing temperature for the part containing the supports was found in the range of 76.3 °C to 225.8 °C, and for the print holder printout itself, the temperature ranged from 179.2 °C to 222.9 °C. The results of the final interpolated temperatures of the printing process of the part and supports are given in Figure 11.

In the next step, the results from the thermal simulation were mapped into mechanical simulation, and the distortion analyses (during printing, after cool down, and after removing supports) were computed. The results of the displacements of the part distortion sequence (after printing, after cool down, and after support removal) are the main outputs of the mechanical analysis. 

**Figure 12:**
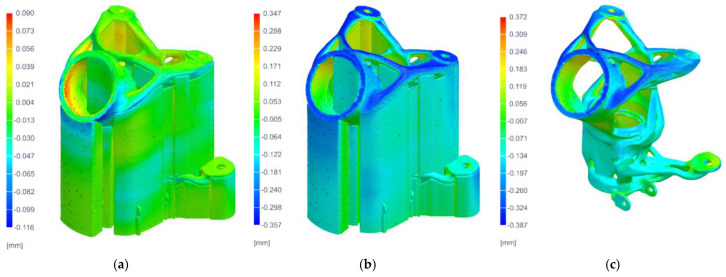
Results of distortion of printed part: (**a**) distortion after printing, (**b**) distortion after cool down, and (**c**) distortion after support removal.

The maximum displacement was logically achieved for the results of the parts after the removal of the supports and reached 0.387 mm (ABS) in the upper area of the print. The graphical results of the part distortion are given in Figure 12.

**Figure 13:**
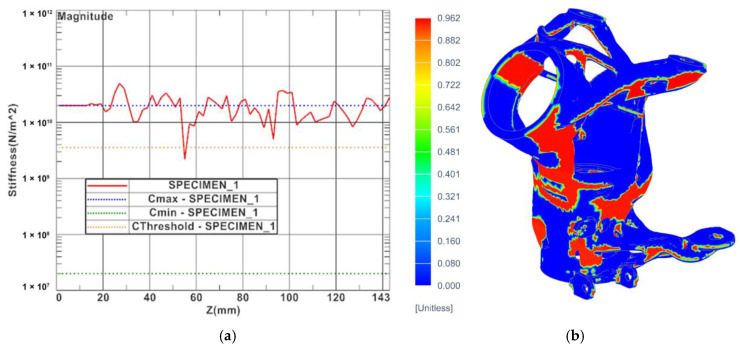
Additional results of numerical simulation: (**a**) stiffness curve of printed part and (**b**) probability of local overheating.

The stiffness curves and local overheating results were generated as additional results of the numerical simulations. The stiffness curve is a 2D curve with layer stiffness information that allows identification of the areas of potential severe distortion or shrink defects. Both results are given in Figure 13.

The last result of the additive manufacturing analysis was checking the possibility of re-coater collision, which also confirmed the suitability of setting up the solution. The input model with compensation from distortion results of the mechanical analysis was exported for fabrication of the function specimen. The distortion compensation was carried out by inverting the distortion prediction and applying the inversion to the nominal target geometry.

### 4.3. Fabrication of Functional Specimen

The functional specimen was fabricated using the industrial 3D printing machine EOS M 290, which uses the L-PBF printing method. The process parameters for printing were chosen the same as for test samples and numerical simulations (see Table 3). The thermal treatment known as ageing was employed during the production of our component using DMLS technology for the AlSi10Mg material. After the AlSi10Mg material was printed, it underwent a natural ageing process that involved heating the component at an elevated temperature followed by gradual cooling. This thermal process enhances the mechanical properties, such as strength and hardness, of the material. The functional specimen of the steering column housing was (after trimming supports) machined in connection areas using a five-axis CNC machine. 

**Figure 14:**
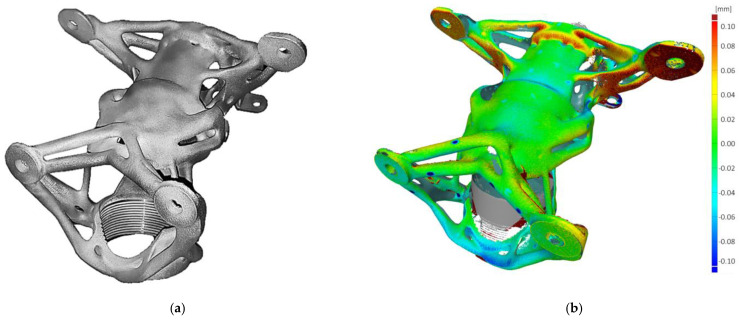
Functional specimen of the steering column housing: (**a**) 3D-printed functional specimen and (**b**) results of deviation.

A 3D scan measurement of the printed functional specimen was performed on a CMM Carl Zeiss Prismo 7 Navigator machine for comparison with results and validation of the compensation (numerical simulation). Very good agreement was found with a deviation in the main functional areas of only up to 0.11 mm. The final functional specimen and comparison of the real state (3D scan) with the original structure of the optimization are shown in Figure 14.

## 5. Discussion

**Figure 15:**
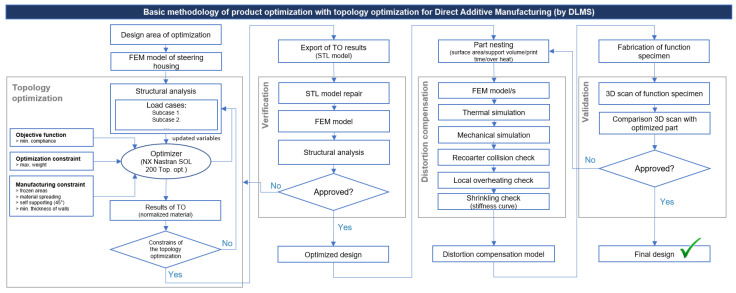
Flowchart of basic methodology of product optimization for direct additive manufacturing by L-PBF.

The successful validation of the proposed methodology for topology optimization and direct additive manufacturing using DMLS was confirmed through extensive testing on the functional sample. The entire process can be divided into four key phases: optimization, verification, distortion compensation, and validation, as depicted in the flowchart illustrated in Figure 15.

To assess the real-world performance of the optimized design, the functional specimen was integrated into the latest generation of the formula student racing car UWB06 and subjected to rigorous testing under actual driving conditions, accumulating over 200 h of active use. These tests closely replicated the forces and stresses predicted by the structural analyses, as determined from telemetry data.

Following the active testing phase, a non-destructive liquid-dye penetrant test was conducted to identify any potential surface cracks on the housing. The results were highly satisfactory, indicating the absence of any detectable flaws. Subsequently, the optimized steering column housing successfully underwent official races without encountering any complications. 

The successful implementation of the proposed methodology, coupled with its impressive performance in real-world racing scenarios, highlights its potential for advancing the field of additive manufacturing and optimization in motorsport applications. These findings pave the way for further exploration and development in the pursuit of enhanced performance and reliability.

The inclusion of fatigue analysis in the methodology, whether through the development of new approaches or the extension of existing ones, presents significant opportunities for further research and advancements in the field. By considering the effects of material fatigue, it opens doors to explore new frontiers and deepen our understanding of component behavior under cyclic loading conditions.

The overall process time consists of three main stages: topology optimization, print simulation, and actual manufacturing. The time savings associated with eliminating the need to export and import data between different stages are 6 h. The time saved by avoiding the need for remodeling is 32 h. Approximately 38 h were saved with the usage of the presented methodology. The total time saved depends on the complexity of the parts, but in general, it can be said that the more complex the design, the greater the amount of time saved.

## 6. Conclusions

The paper presents a novel methodology for utilizing the results of topology optimization directly in additive manufacturing (L-PBF) without the need for remodeling the final structure. A case study involving the steering column housing exemplifies the application of this process. The optimal shape was obtained using topology optimization in Siemens NX 1888 Software, with consideration of additive manufacturing constraints. To verify the suitability of the resulting structure, the normalized material data from topology optimization were converted into a convergent body for verification analysis. The FEM-based solver NX Nastran verified the obtained design. Furthermore, numerical simulations of the printing process were conducted to address potential printing issues such as thermal distortion, overheating areas, and shrinking lines.

The proposed methodology demonstrated significant time savings in the overall design process, particularly by eliminating the need for remodeling the resulting structure obtained from topological optimization. However, this advantage is simultaneously associated with a major limitation of the proposed methodology. As the obtained structure is very challenging to edit, it becomes highly problematic to modify the final geometry in terms of functionality, especially if it does not fully meet the requirements after verification analysis. Furthermore, applying shape optimization to the remodeled structure easily resolves critical areas with high stress concentrations.

To validate the methodology, a functional specimen of the steering column housing was fabricated using AlSi10Mg on an EOS M 290 3D printer. The printed part was then 3D-scanned to assess distortion, and it underwent extensive testing during a full season in the single-seat formula student racing car UWB06. Remarkably, the component endured over 200 h of active driving in both testing and official races without any complications.

## Figures and Tables

**Table 1:** Chemical composition of AlSi10Mg (wt.%).

Al	Si	Mg	Fe	Ti	Cu	Mn	Zn
Balanced	9.9	0.43	0.24	<0.01	<0.005	<0.005	<0.002

**Table 2:** Process parameters of AlSi10mg specimens.

Power (W)	Scan Rate (mm/s)	Layer Thickness (µm)	Hatch Distance (µm)	Platform Temperature (°C)
370	1150	30	100	150

**Table 3:** Mechanical properties of AlSi10Mg (EOS M 290) of the vertically and horizontally printed specimens.

Parameter	Print Direction		Description
	VPS	HPS	
ρ (kg/m^3^)	2670		Density
E (MPa)	70	69	Modulus of elasticity
ν (-)	0.29		Poisson’s ratio
Rp0.2% (MPa)	192	224	Yield strength
$\sigma_{k}$ (MPa)	468	471	Tensile strength
$\varepsilon_{k}$ (%)	9 ± 2	11 ± 2	Elongation at break

**Table 4:** Process/thermal parameters of AlSi10Mg (EOS M 290) used for numerical simulation.

Parameter	Value	Description
t_R_ (s)	12.25	Recoating time
T_BP_ (°C)	147	Base-plate temperature
T_A_ (°C)	46.85	Ambient temperature
h_HTC_ (W/(m.K))	20	Ambient Gas HT
α_BP_ (°C^−1^)	1.20^e−05^	Build plate/printer alpha
n_PL_	1	Number of lasers of printer

## Data Availability

Not applicable.
